# The Work Gratitude Scale: Development and Evaluation of a Multidimensional Measure

**DOI:** 10.3389/fpsyg.2021.795328

**Published:** 2022-01-05

**Authors:** Carolyn M. Youssef-Morgan, Llewellyn E. van Zyl, Barbara L. Ahrens

**Affiliations:** ^1^College of Business, Bellevue University, Bellevue, NE, United States; ^2^Department of Industrial Engineering and Innovation Sciences, University of Eindhoven, Eindhoven, Netherlands; ^3^Optentia Research Unit, North-West University, Vanderbijlpark, South Africa; ^4^Department of Human Resource Management, University of Twente, Enschede, Netherlands; ^5^Department of Social Psychology, Institut für Psychologie, Goethe University, Frankfurt am Main, Germany; ^6^Waukee Community School District, Waukee, IA, United States

**Keywords:** gratitude, work gratitude, positive psychology, positive organizational behavior, scale development, measurement, positive psychological assessment

## Abstract

This study explores gratitude as a multidimensional and work-specific construct. Utilizing a sample of 625 employees from a variety of positions in a medium-sized school district in the United States, we developed and evaluated a new measure, namely the Work Gratitude Scale (WGS), which encompasses recognized conative (intentional), cognitive, affective, and social aspects of gratitude. A systematic, six-phased approach through structural equation modeling (SEM) was used to explore and confirm the factorial structure, internal consistency, measurement invariance, concurrent, convergent, and discriminant validity of the WGS. The results supported a 10-item measure with three dimensions: “grateful appraisals” (three items), “gratitude toward others” (four items), and “intentional attitude of gratitude” (three items). Thereafter, first-order, second-order, and bifactor confirmatory models were estimated and compared. Work gratitude was found to be best described by a second-order construct with three underlying first-order dimensions. Measurement invariance was supported in relation to gender. Concurrent validity was supported in relation to two existing dispositional gratitude scales, namely the Gratitude Questionnaire and the Gratitude, Resentment, and Appreciation Scale (GRAT). Convergent validity was supported in relation to the Core Self-Evaluations Scale (CSES) and the Psychological Capital Questionnaire. Discriminant validity was supported in relation to various demographic factors such as age, gender, occupation, and tenure. The findings support the WGS as a multidimensional measure that can be used in practice to measure overall work-related gratitude and to track the effectiveness of gratitude-related workplace interventions.

## Introduction

As both a state and a psychological strength, gratitude has become one of the fundamental building blocks of positive psychology (van Zyl et al., 2021). Emmons (2004) defines gratitude as “a sense of thankfulness and joy in response to receiving a gift, whether the gift can be a tangible benefit from a specific other or a moment of peaceful bliss evoked by natural beauty” (p. 554). There are many recognized benefits of gratitude. For example, gratitude promotes health, wellbeing and life satisfaction (Dickens, 2017). Gratitude is also related to effective coping (Wood et al., 2007), development of social support, and reduction of stress and depression (Wood et al., 2008a). Additionally, gratitude leads to prosocial behaviors that can promote healthy, satisfying, and productive relationships and connections (Portocarrero et al., 2020).

To-date, with very few exceptions, much of the empirical gratitude research pertains to non-work domains (Emmons, 2004; Watkins, 2014). On the other hand, in the context of positive organizational behavior, Luthans et al. (2015) propose gratitude as an evidence-based positive psychological resource that is open to development and management in the workplace. Relatedly, Fehr et al. (2017) emphasize that “organizations are not simply extensions of everyday social interactions. Rather, the organizational context introduces a unique set of constraints and affordances that influence how individual employees feel, think, and act on a daily basis” (p. 361) and make a case for a multifaceted work gratitude model that incorporates several personal, situational, and organizational contingencies. It follows that work gratitude may also exhibit unique dimensionality that may necessitate the purposeful design and validation of dedicated measures. This study seeks to fill this gap, by developing a work-specific and multidimensional measure of gratitude.

### Conceptualizations of Gratitude

Gratitude has been conceptualized and measured as a *general, stable dispositional trait* (Portocarrero et al., 2020) or an enduring virtue (Peterson and Seligman, 2004; Morgan et al., 2017). Seligman et al. (2005) conceptualize character strengths such as gratitude as “trait-like—an individual difference with demonstrable generality and stability” (p. 411). In other words, within this perspective, grateful people have a general tendency to experience thankfulness or appreciation more frequently, more intensely, for longer periods, and across a broader range of people and situations than their less grateful counterparts (McCullough et al., 2002). In the context of the workplace, Cain et al. (2019) conceptualize gratitude as a dispositional trait or an enduring “tendency to notice and be thankful for how various aspects of a job affect one’s life” (p. 441).

However, alternative, more malleable perspectives of gratitude also abound and are supported by abundant empirical evidence. Gratitude has been shown to involve several cognitive, affective, social, and situational factors (Wood et al., 2008b,^2009^). Research shows that gratitude varies with situations and events (Wood et al., 2008a). Gratitude can also change over time (Froh et al., 2010; Chopik et al., 2019). Importantly, gratitude is open to development through a variety of relatively simple and practical interventions (*c.f.*, Dickens, 2017; Richter et al., 2021). Interestingly, recent studies utilizing functional neuromagnetic imaging (fMRI) provide tangible neuroimaging evidence that gratitude, previously thought to be a “hardwired” tendency, is indeed malleable and responsive to basic, short, well-recognized development interventions such as gratitude journaling (Karns et al., 2017). Thus, gratitude cannot be just a general, dispositional trait.

Within the malleable perspectives of gratitude, at least four views can be found: (a) affective/emotional, (b) cognitive/evaluative, (c) social/other-focused, and (d) conative/intentional. First, gratitude has been supported as a quick, intense, and constantly fluctuating *emotional state*, with substantial within-person variability (Emmons and Mishra, 2011; Spence et al., 2014). Second, gratitude can be viewed as a *situation-specific cognition* that involves positive appraisals of various aspects of a particular situation. For example, according to Emmons and Crumpler (2000), gratitude is the appreciation of an altruistic gift. This appreciation requires recognizing the gift, recognizing the goodness of the gift, recognizing the goodness of the giver, and recognizing the benefits of the gift that go beyond one’s social expectations of others (Watkins, 2014). In other words, the extent of gratitude one experiences is often dependent on a combination of perceptions and evaluations of the value of the gift to the recipient, the cost of the gift to the giver, and the benevolence (i.e., altruistic intentions) of the giver toward the recipient of the gift. This multifaceted cognitive perspective goes beyond both the enduring trait and the purely transient affective state conceptualizations of gratitude.

Third is the *social/other-focused* perspective. Conceptualizations of this perspective vary in the literature along several dimensions. As indicated in the opening definition, the object of gratitude may another person’s generosity, or it can be a less tangible “moment” of thankfulness or appreciation of a specific event, or of one’s blessings and fortune in general (Emmons, 2004). When the object of gratitude is another person’s actions, another social dimension of gratitude is whether it involves reciprocating the gift or benevolent act. Furthermore, if gratitude involves reciprocity, the next social dimension is whether reciprocity is directed toward the original giver or another recipient, often referred to as “paying it forward” or prosocial behaviors in general (*c.f.*, Ma et al., 2017).

The fourth and final perspective is the *conative/intentional* view of gratitude. In this view, a distinction is made between reactive gratitude, in which a person may experience gratitude as a result of the benevolence of others or being overwhelmed by life’s abundant blessings, and a proactive attitude, which is more intentional in nature. Being proactively grateful entails consciously choosing to be grateful and intentionally finding ways to do so. Examples of intentional gratitude include purposefully counting one’s blessings, enjoying life’s simple pleasures, and stopping to smell the roses (Watkins et al., 2003). Indeed, the most recognized gratitude development interventions closely follow these purposeful and intentional practices (Seligman et al., 2005).

Importantly, the above four perspectives or views of gratitude can and do overlap. For example, cognitive appraisals and conative aspects of gratitude may be necessary to determine social reciprocity actions, and all views of gratitude are likely to produce positive emotions. Thus, an integrative and multidimensional perspective of gratitude is both necessary and more accurate than narrower definitions and conceptualizations (Morgan et al., 2017). Furthermore, synergistic integration of these four perspectives (affective, cognitive, social, and conative) has also been conceptually supported in the context of similar positive psychological resources, such as psychological capital (PsyCap), a higher order construct that includes hope, efficacy, resilience, and optimism (Youssef and Luthans, 2013; Youssef-Morgan and Luthans, 2013; Luthans and Youssef-Morgan, 2017).

Integrating this four-pronged conceptualization of gratitude, we define *work gratitude* as “the intentional choice to engage in positive appraisals and feelings of thankfulness and appreciation toward the characteristics, situations, and people currently present in one’s work context.” Specifically, this definition synthesizes the conative (intentional choice), cognitive (positive appraisals), affective (feelings), and social (people) aspects of gratitude. Further, it takes into consideration that gratitude is a situational and context-specific state, rather than just a general disposition.

### Relevant Approaches for Measuring Work Gratitude

The most commonly used measures of general gratitude are the *Gratitude Questionnaire* (GQ6; McCullough et al., 2002) and the *Gratitude, Resentment, and Appreciation Scale* (GRAT; Watkins et al., 2003). The GQ6 is a six-item scale that measures dispositional gratitude. Although dispositional gratitude is conceptualized in terms of frequency, intensity, span, and density, this scale assesses dispositional gratitude as a unidimensional factor (McCullough et al., 2002).

The GRAT also measures dispositional gratitude (Watkins et al., 2003). However, this 44-item scale, or its 16-item short version (Thomas and Watkins, 2003; Diessner and Lewis, 2007), are both multidimensional. They measure trait gratitude along three dimensions: sense of abundance (lack of sense of deprivation), simple appreciation (appreciation for simple pleasures), and appreciation for others (social appreciation).

Gratitude has also been measured as an affective state (Spence et al., 2014). This five-item scale measures state gratitude as a unidimensional, discrete emotion. While Spence et al. (2014) study is work-related (linked gratitude to organizational citizenship behaviors), the scale they developed is an measure of state gratitude in general, not work gratitude. Gratitude has also been conceptualized and measured as a multidimensional moral virtue (Morgan et al., 2017), with dimensions somewhat consistent with earlier views.

Recent studies demonstrate the relevance of gratitude in the workplace, both conceptually and empirically (Spence et al., 2014; Luthans et al., 2015; Fehr et al., 2017; Cain et al., 2019). However, measures of gratitude as a work-specific construct are lacking. A notable exception is Cain et al.’s (2019) recently developed Gratitude at Work Scale, which measures gratitude as a dispositional trait along two dimensions: gratitude for supportive work environment, and gratitude for meaningful work. While these two dimensions are highly relevant, we believe that they do not encompass the full range of conative, cognitive, affective, and social aspects of gratitude. Importantly, conceptualizing and measuring work gratitude as a dispositional trait or enduring tendency contradicts the extensive literature supporting the efficacy of short and simple gratitude interventions in changing participants’ gratitude levels (including Study 3 of Cain et al., 2019), which point to the malleability and state-like nature of gratitude (Luthans et al., 2015). Because the scale focuses on gratitude as a dispositional trait, it does not cover the volitional or intentional aspects of gratitude. It simply asks participants to recount how often they are grateful for various aspects of the workplace (e.g., coworkers, supervisors, clients, salary and benefits, work-life balance, autonomy, accomplishments, and growth opportunities).

We draw from the PsyCap literature, where a multidimensional work-specific measure of a positive psychological construct was developed and validated. PsyCap is supported as a higher-order construct, with hope, efficacy, resilience, and optimism as lower-order constructs. The Psychological Capital Questionnaire (PCQ-24, Luthans et al., 2007) was developed by adapting items from dispositional measures of hope, efficacy, resilience, and optimism. The wording of each item was adapted in two distinct ways. First, because the original scales were designed to measure the trait counterparts of each resource, they were adapted by adding language that reflects the state-like, “here and now” timeframe typically experienced in the work context. This approach was also used by Snyder et al. (1996) in adapting the dispositional hope scale (Snyder et al., 1991) to create a state hope scale. Examples of language added include: “at the present time,” “currently,” and “right now.” Second, with the exception of efficacy, the original scales were designed to measure general, not context-specific psychological constructs. Thus, the words “work,” “at work,” or “as it pertains to my work” were added to each item to make it work-specific.

Subsequently, a shorter, 12-item version (PCQ-12) of the PCQ-24 was developed (Avey et al., 2011). The PCQ-12 became widely used, particularly in cross-cultural research due to the reduced cost and increased ease of translation. One of the most notable changes is that the PCQ-12 does not include any reverse-scored items. Research shows that reverse-scored and negatively worded items tend to form a separate factor and change the dimensionality of a construct (Tomas and Oliver, 1999). Also they tend to lower the reliability of multi-item scales by as much as 20% (Barnette, 2000). This problem is particularly prevalent in measures of positive constructs, because seemingly opposite positive and negative constructs may not necessarily be opposite ends of a single construct, but rather distinct constructs (Peterson and Chang, 2002; Merritt, 2012). These method effects are evident in investigations of a number of established scales (DiStephano and Motl, 2006; Salazar, 2015). Thus, there is an abundance of evidence against the use of reverse-scored or negatively worded items.

The PCQ-12 was further adapted to other contexts beyond the workplace, such as health PsyCap, relationships PsyCap, overall (life in general) PsyCap (Luthans et al., 2013), and academic PsyCap (Martínez et al., 2019, 2021). Adaptation to most contexts is relatively easy. It can be accomplished simply by replacing the word “work” with the desired context. Context adaptations were not found to compromise the validity, reliability, or dimensionality of the scale.

Based on the lessons learned from the development and adaptation of PsyCap measures, it follows that a plausible approach to developing, or at least generating an initial list of items for, a work gratitude scale (WGS) is to adapt the items in existing trait gratitude scales. This can be accomplished by adding wording to reflect (a) the “here and now” timeframe and (b) the “at work” context. Additionally, (c) the adapted items should be positively worded, rather than reverse-scored.

With this approach in mind, the purpose of this study was to develop, evaluate and validate a robust measure for work gratitude. Specifically, it aimed to explore the factorial structure, internal consistency, measurement invariance (gender), concurrent, convergent and discriminant validity of the WGS.

## Materials and Methods

### Research Approach

A cross-sectional survey-based research design was utilized to collect the data for this study.

### Participants

A convenience sample of 625 participants volunteered to take part in the study. Participants held a variety of positions in a medium-sized school district in the United States. At the time of the study, the school district employed about 900 employees, all of whom were invited to participate. Table 1 provides a descriptive overview of the demographic characteristics of the sample.

**TABLE 1:** Demographic characteristics of participants (*N* = 625).

Item	Category	Frequency	Percentage
		(*f)*	(%)
Gender	Male	108	17.3
	Female	514	82.2
	Missing	3	0.5
Age	18–20	0	0
	21–25	122	19.5
	26–30	196	31.4
	31–35	177	28.3
	36–40	112	17.9
	41–50	17	2.7
	51+	0	0
Tenure (years)	0–5	262	41.9
	6–10	153	24.5
	11–15	98	15.7
	16–20	59	9.4
	21+	53	8.5
Occupation	Leadership team	43	6.88
	Administrative support staff	44	7.04
	Teacher	424	67.84
	Associate	58	9.28
	Food preparation and serving	12	1.92
	Building/grounds/maintenance	5	0.8
	Other	39	6.24

### Measures

#### The Work Gratitude Scale

The WGS was developed based on the approach employed by Luthans et al. (2007), by adapting existing trait measures of positive psychological constructs to the present, “here and now,” and “at work” context. We also consulted recommended assessment approaches to-date for state and context-specific gratitude (McCullough et al., 2002; Wood et al., 2008a; Watkins et al., 2003), and heeded advice regarding avoiding negatively worded (reverse-scored) items, particularly when measuring positive psychological constructs (Tomas and Oliver, 1999; Barnette, 2000; Peterson and Chang, 2002; Merritt, 2012).

The initial pool of items was drawn from the GQ-6 (6 items) and the GRAT short version (16 items), which yielded 22 items for further consideration. Seven items were reverse-scored (negatively worded) and thus excluded, reducing the number to 15 items. Two items of the GRAT could not be adapted to the work context (“oftentimes I have been overwhelmed at the beauty of nature” and “every Fall I really enjoy watching the leaves change colors”). Thus, 13 items were adapted and used on the survey. Before survey administration, the 13 items were reviewed by an expert panel of positive psychology scholars to assess face validity and for final fine-tuning. The full list of items is shown in Table 4 and discussed further in subsequent sections. Each item was rated on a seven-point Likert scale ranging from 1 (Strongly Disagree) to 7 (Strongly Agree). The final measure was comprised of 10 items (*c.f.*, Appendix A).

**TABLE 2:** Model fit statistics.

Fit indices	Cut-off criterion	Sensitive to *N*	Penalty for model complexity
***Absolute fit indices***			
Chi-square (χ^2^)	• Lowest comparative value between measurement models	Yes	No
	• Non-significant Chi-square (*p* > 0.01)		
	• Significant difference in Chi-square between models		
	• For model comparison: retain model with lowest Chi-square		
χ^2^/*df*	•<3 = Excellent and <5 = Acceptable	No	No
	• For model comparison: retain model with Δχ^2^/df > 1		
***Approximate fit indices***			
Root-Means-Square Error of Approximation (RMSEA)	• 0.06–0.08 (Marginally acceptable); 0.01–0.05 (excellent)	No	Yes
	• Not-significant (*p* > 0.01)		
	• 90% confidence interval range should not include zero		
	• For model comparison: retain model where ΔRMSEA ≤ 0.015		
Standardized Root Mean Square Residual (SRMR)	• 0.06–0.08 (Marginally acceptable); 0.01–0.05 (excellent)	Yes	No
	• For model comparison: retain model where ΔSRMR ≤ 0.015		
***Incremental fit indices***			
Comparative Fit Index (CFI)	• 0.90–0.95 (Marginally acceptable fit); 0.96–0.99 (excellent)	No	No
	• For model comparison: retain model with highest CFI value (ΔCFI > 0.01)		
Tucker-Lewis Index (TLI)	• 0.90–0.95 (Marginally acceptable fit); 0.96–0.99 (excellent)	No	Yes
	• For model comparison: retain model with highest TLI value (ΔTLI > 0.01)		
Akaike Information Criterion (AIC)	• Lowest value in comparative measurement models	Yes	Yes
Bayes Information Criterion (BIC)	• Lowest value in comparative measurement models	Yes	Yes

*Adapted from Hu and Bentler (1999) and Wong and Wong (2020).*

**TABLE 3:** Model fit statistics for competing exploratory factorial models.

Model	Type	χ^2^	*df*	χ^2^/*df*	CFI	TLI	RMSEA		SRMR	AIC	BIC	aBIC	Meets criteria
Model 0	One factor model	1365.23	65.00	21.00	0.78	0.74	0.18	[0.172–0.189]	0.09	19147.92	19320.36	19196.54	No
Model 1	Two factor model	744.81	53.00	14.05	0.88	0.83	0.15	[0.137–0.155]	0.06	18551.50	18777.01	18615.09	No
Model 2	Three factor model	188.791	42.00	4.50	0.98	0.95	0.08	[0.065–0.086]	0.02	18017.48	18291.62	18094.78	Partially
Model 3	Three factor model (removed items)	**23.45**	18.00	1.30	1.00	1.00	**0.02**	[0.000–0.045]	0.01	14260.60	14468.57	14319.35	Yes

*χ^2^ = Chi-square; df = degrees of freedom; TLI = Tucker-Lewis Index; CFI = Comparative Fit Index; RMSEA = Root Mean Square Error of Approximation [90% CI]; SRMR = Standardized Root Mean Square Residual; AIC = Akaike Information Criterion; BIC = Bayes Information Criterion; aBIC = Adjusted Bayes Information Criterion.*

*Bold: Non-significant p > 0.001.*

**TABLE 4:** Exploratory factor analysis-factor loadings and variance.

Label	Item	EFA Model 2				EFA Model 3 (removed items)			
		λ*_1_*	λ*_2_*	λ_*3*_	*R*^2^ (%)	λ*_1_*	λ*_2_*	λ_*3*_	*R*^2^ (%)
***Grateful appraisals***									
WGS 1	Right now, I have so much at work to be thankful for.	**0.91**	−0.06	0.02	58.23	**0.94**	−0.06	−0.01	58.19
WGS 2	At this present time, if I had to list everything that I felt grateful for at work, it would be a very long list.	**0.81**	0.04	0.06	–	**0.83**	0.05	0.04	–
WGS 6	At the present time, life has been good to me at work.	**0.71**	0.12	0.01	–	**0.69**	0.12	0.00	–
***Gratitude toward others***									
WGS 5	Currently, I couldn’t have gotten where I am today at work without the help of many people.	−0.05	**0.84**	−0.05	9.59	−0.03	**0.81**	−0.04	11.67
WGS 7	Although I think it’s important to feel good about my current work accomplishments, I think that it’s also important to remember how others have contributed to my accomplishments.	0.05	**0.85**	−0.01	–	0.08	**0.85**	−0.03	–
WGS 8	Although I’m basically in control of my work at the present time, I can’t help but think about all those who have supported me and helped me along the way.	−0.07	**0.86**	0.10	–	−0.05	**0.89**	0.06	–
WGS 12	Right now, I feel deeply appreciative for the things others have done for me at work.	0.19	**0.51**	0.17	–	0.19	**0.49**	0.17	–
***Intentional attitude of gratitude***									
WGS 9	Currently, I think that it’s important to “Stop and smell the roses” as it pertains to my work.	−0.10	0.04	**0.82**	7.82	−0.09	0.04	**0.82**	9.67
WGS 10	Currently, I believe that it’s important to pause often to “count my blessings” at work.	0.06	0.06	**0.83**	–	0.09	0.07	**0.80**	–
WGS 11	Right now, I think it’s important to enjoy the simple things that pertain to my work.	0.02	−0.06	**0.88**	–	0.03	−0.07	**0.89**	–
***Double loadings***									
WGS 3	At this time, I am grateful to a wide variety of people at work.	**0.38**	**0.47**	−0.01	–	–	–	–	–
WGS 4	Right now, I find myself able to appreciate the people, events, and situations that have been part of my work history.	**0.35**	**0.44**	0.05	–	–	–	–	–
WGS 13	Today, I believe it’s important to appreciate each day at work.	**0.36**	0.07	**0.50**	–	–	–	–	–

*λ = Factor Loading; R^2^ = Variance. Bold = Significant loading with p < 0.01.*

#### Concurrent Validity Measures

The GQ6 was used to measure the frequency, intensity and density of dispositional gratitude (McCullough et al., 2002). The six item, self-report questionnaire assesses dispositional gratitude as a unidimensional factor and is rated on a seven-point Likert type scale ranging from 1 (“Strongly Disagree”) to 7 (“Strongly Agree”). A sample item is “I am grateful to a wide variety of people.” The GQ-6 has shown to be a reliable measure in various contexts with an average Cronbach of 0.81 across 58 samples (Card, 2019).

The GRAT was used to measure dispositional gratitude as a stable trait (Watkins et al., 2003). The 16-item GRAT short version (Thomas and Watkins, 2003; Diessner and Lewis, 2007) is a multidimensional measure that assesses three components of gratitude: sense of abundance [lack of sense of deprivation. e.g., “There never seems to be enough to go around and I never seem to get my share (r)”], simple appreciation (appreciation for simple pleasures, e.g., “Oftentimes I have been overwhelmed at the beauty of nature”), and appreciation for others (social appreciation; e.g., “I feel deeply appreciative for the things others have done for me in my life”) on a nine-point Likert scale ranging from 1 (“Strongly Disagree”) to 9 (“Strongly Agree”). A meta-analysis has shown that the GRAT is a reliable measure in various contexts with an average Cronbach of 0.92 across 5 samples (Card, 2019).

#### Convergent Validity Measures

The Core Self-Evaluations Scale (CSES) was used to measure participants overall perception of self (Judge et al., 2003). The 12-item scale is a higher-order construct that measures four established personality traits: self-esteem (“Overall, I am satisfied with myself”), generalized efficacy (e.g., “I am confident I get the success I deserve in life”), locus of control (“I determine what will happen in my life”), and neuroticism (“There are times when things look pretty bleak and hopeless to me”). The CSES measures these as a unidimensional construct. Each item is rated on a five-point Likert type scale ranging from 1 (“Strongly Disagree”) to 5 (“Strongly Agree”). The instrument has shown to have high levels on internal consistency with Cronbach Alphas greater than 0.80 and test-retest reliability of 0.81 (Gardner and Pierce, 2010).

The PCQ-12 was used to measure PsyCap (Avey et al., 2011). The 12-item scale measures a higher-order psychological capital construct that is comprised of four first order factors: hope (e.g., “Right now I see myself as being pretty successful at work”), efficacy (e.g., “I feel confident in representing my work area in meetings with management”), resilience (e.g., “I usually take stressful things at work in stride”), and optimism (e.g., “I always look on the bright side of things regarding my job”). Each item is rated on a six-point Likert type scale ranging from 1 (“Strongly Disagree”) to 7 (“Strongly Agree”). The questionnaire has shown to be a reliable measure with McDonalds Omega’s ranging from 0.72 to 0.90 on the various sub-scales (Rice et al., 2021).

#### Discriminant Validity Measures

Discriminant validity was assessed by relating the WGS to various demographic factors such as age (in years), gender, type of occupation, and tenure (in years).

### Statistical Analyses

Data were analyzed using SPSS v26 (IBM SPSS, 2019), JASP v. 0.14.1 (JASP, 2021), and Mplus v 8.6 (Muthén and Muthén, 2021). A systematic, six-phased approach through structural equation modeling (SEM) was used to determine the factorial validity, internal consistency, measurement invariance with gender, concurrent, convergent and discriminant validity of the WGS.

*First*, to explore the factorial structure and item loadings of the WGS, an exploratory factor analytical (EFA) approach through SEM was employed. To determine the factorability of the instrument, the Kaiser-Meyer-Olkin (KMO) approach and Bartlett’s sphericity test were estimated. According to Kaiser and Rice (1974), both a KMO value larger than 0.60 and a significant chi-square on Bartlett’s sphericity assessment would indicate that meaningful factorial structures could be extracted from the data. Thereafter, a competing EFA modeling approach with the maximum likelihood estimation (ML) method, and a direct Oblimin rotation was used through the SEM framework. Here, competing exploratory factorial models were specified to be extracted from the data, based on Eigenvalues larger than 1 (Muthén and Muthén, 2021). Competing EFA models were compared based on model fit statistics with associated cut-off criteria (*c.f.*, Table 2), items were required to load statistically significantly (factor loading > 0.40; *p* < 0.05) on their respective extracted factors, cumulatively all factors needed to declare at least 50% total variance, and items should not represent multiple factors. Dual loadings were systematically removed, and models were re-estimated (Wong and Wong, 2020).

*Second*, the factorial validity of the WGS was explored. Based on the best fitting EFA model, a competing confirmatory factor analytical measurement modeling strategy with the ML estimator was used to explore different, theoretically informed factorial permutations of the WGS. Both traditional independent cluster modeling confirmatory factor analytical (ICM-CFA) models and a bifactor model were estimated and sequentially compared. Observed items were used as indicators for latent factors. Observed indicators were only permitted to load onto their *a priori* theoretical factors and cross-loadings were not permitted. For the bifactor model, a single general factor (Work Gratitude) and three specific factors (“grateful appraisals,” “gratitude toward others,” and “intentional attitude of gratitude”) were specified. All items were estimated to load on the general factor. For the specific factors, items were targeted to load onto their *a priori* factors. Here an orthogonal targeted rotation was used, and all covariances between specific factors were constrained to zero. To determine the best fitting model for the data, both the Hu and Bentler’s (1999) criteria for model fit indices (*c.f.*, Table 2 for the criteria) as well as indicators of measurement quality were employed. Measurement quality for the best fitting measurement models was assessed through evaluating the standardized factor loadings (λ > 0.40; *p* < 0.05), item uniqueness (>0.1 but <0.9; *p* < 0.05), and the presence of no multiple cross-loadings (Kline, 2010). Models that showed both excellent fit and measurement quality were retained for further analyses (McNeish et al., 2018).

*Third*, for the best fitting traditional ICM-CFA measurement models, item-level descriptive statistics (means, standard deviations, skewness, and kurtosis), standardized factor loadings, corrected item-total correlations (CITC), average variance extracted (AVE), and levels of internal consistency were estimated. To determine the multivariate normality of each item, Kim (2013) suggested that absolute values for skewness (<2) and kurtosis (<2) be employed for samples larger than 500. CITC represents each individual item’s relationship to the overall factor on which it loads (Zijlmans et al., 2019). A CITC lower than *r* = 0.30 indicates that a specific item might not accurately represent the overall factor on which it is specified (Zijlmans et al., 2019). To determine the level of internal consistency of the WGS, three indicators were computed: point-estimate composite reliability (upper-bound; ρ > 0.80; Raykov, 2009), McDonald’s Omega (ω > 0.80; Hayes and Coutts, 2020), and Cronbach’s alpha (lower-bound; α > 0.70; Nunnally and Bernstein, 1994). Further, AVE was used as an indicator of the average level of reliability of each item within the scale. Here, levels of 50% or higher are deemed acceptable (Kline, 2010).

Additionally, for the bifactor model, the explained common variance (ECV), the item level explained common variance (IECV), the Average Relative Parameter Bias (ARPB), as well as Omega_s_ for the specific factor (ω_specific_ > 0.80) and Omega_h_ (ω_hierarchical_ > 0.80) for the general factor as indicators of reliability were computed. ECV refers to the proportion of total common variance explained by a general factor within bifactor models (Stucky and Edelen, 2015). For the specific factors, ECV represents the strength of the factor relative to all the explained variance within each specific factor. An ECV value for the general factor should exceed 0.50 (Stucky et al., 2013). Similarly, the IECV refers to “the extent to which an item’s responses are accounted for by variation on the latent general dimension alone, and thus acts as an assessment of unidimensionality at the individual item level” (Stucky et al., 2013, p. 51) which should also exceed 0.50. ARPB represents an indicator of item bias within bifactor models. Here an ARPB value of less than 0.15 (i.e., 15%) is acceptable and therefore “poses no serious concern” (Rodriguez et al., 2016).

*Fourth*, we also investigated the factor equivalence or “measurement invariance” for gender (males vs. females). Here, increasingly restrictive equality constraints were placed on the best fitting measurement models. Configural (similar factor structures), metric (similar factor loadings) and scalar (similar intercepts) invariance models were estimated and sequentially compared. Invariance was established when the following criteria are met: (a) a non-significant difference in χ^2^ between increasingly restrictive models, and (b) non-significant changes in RMSEA (Δ < 0.015), SRMR (Δ < 0.015), CFI (<0.01), TLI (<0.01), and χ^2^/*df* (<1) between increasingly constrained models (Cheung and Rensvold, 2002; Reise et al., 2013). Once invariance was established, we proceeded to estimate latent mean differences between males and females. Here, the latent mean score for the reference group (males) was constrained to zero. The mean score for the female group was then freely estimated. If the latent mean score differed significantly (*p* < 0.05) from zero, it would indicate meaningful differences between genders (Wickrama et al., 2016; Wong and Wong, 2020).

*Fifth*, to establish convergent validity, the best fitting models of the WGS were then entered into a measurement model with both the GQ6 and the GRAT scales. The measurement models needed to fit the data based on the criteria in Table 3, as well as produce a statistically significant (*p* < 0.05) standardized correlation between WGS and the GQ6 and GRAT scales (Kline, 2010).

*Finally*, to investigate concurrent validity and discriminant validity, structural models were used. Here two separate structural models were estimated for the ICM-CFA and bifactor models. For concurrent validity, the WGS (as an exogenous factor) was regressed on the CSES and the PCQ-12 (as endogenous factors). We established discriminant validity within the same structural model by estimating correlations between the WGS and predefined demographic factors (gender, age, occupation, and tenure). For the bifactor model, a similar approach was employed, however, both the general factor as well as the three specific factors were estimated to be related to the endogenous factors and correlated with the demographic factors. A significance level of *p* < 0.05 (95% confidence interval) was set for each regressive path and correlation.

## Results

### Exploratory Factor Analysis

An EFA approach was employed to explore the factorial structure of the WGS. First, the KMO measure and Bartlett’s test for sphericity were used to determine the factorability of the instrument. Results showed that meaningful factors could be extracted from the data because the KMO value was larger than 0.60 (KMO = 0.93) and a significant chi-square [χ^2^_(625)_ = 5996.35, *df* = 78, *p* < 0.01] was produced. We therefore proceeded to estimate the EFA models in Mplus.

Initially five factorial models were specified to be extracted from the data. Results showed that three factors could be extracted with eigenvalues larger than 1. Further, only three of the five models converged. Therefore, only the one, two, and three factorial models could be inspected (*c.f.*, Table 3). EFA Model 0, a *single first order factorial model* [χ^2^_(615)_ = 1365.22; *p* < 0.01; *df* = 65; χ^2^/*df* = 21.00; CFI = 0.78; TLI = 0.74; RMSEA = 0.18 [0.172, 0.189], *p* < 0.01; SRMR = 0.09; AIC = 19147.92; BIC = 19320.36; Eigenvalue = 7.57; *R*^2^ = 58.23%] did not fit the data and was therefore rejected. EFA Model 1, a *two first order factorial model* [χ^2^_(615)_ = 744.814; *p* < 0.01; *df* = 53; χ^2^/*df* = 14.05; CFI = 0.88; TLI = 0.83; RMSEA = 0.15 [0.137, 0.155], *p* < 0.01; SRMR = 0.06; AIC = 18551.50; BIC = 18777.01; Eigenvalue Factor 1 = 7.57; *R*^2^ = 58.23%; Eigenvalue Factor 2 = 1.25; *R*^2^ = 11.67%], also did not fit the model fit criteria, and was also rejected. Only EFA Model 2, the *three first order factorial model* [χ^2^_(615)_ = 188.79; *p* < 0.01; *df* = 42; χ^2^/*df* = 4.95; CFI = 0.98; TLI = 0.95; RMSEA = 0.08 [0.065, 0.086], *p* < 0.01; SRMR = 0.02; AIC = 18017.48; BIC = 18291.62; Eigenvalue Factor 1 = 7.57; *R*^2^ = 58.23%; Eigenvalue Factor 2 = 1.25; *R*^2^ = 11.67%; Eigenvalue Factor 3 = 1.02; *R*^2^ = 7.82%], fitted the data the best.

The three-factor EFA Model 3 also showed to fit the data significantly better than the one and two first-order factorial models. The item loadings and declared variance for this model are presented in Table 4. The results showed that three items (WGS3, WGS4, and WGS13) needed to be removed due to dual loadings, and therefore the three-factor model was respecified to produce new fit statistics. EFA Model 3, the *three first order factorial model with the three items removed* [χ^2^_(615)_ = 23.45; *p* > 0.01; *df* = 18; χ^2^/*df* = 1.30; CFI = 1.00; TLI = 1.00; RMSEA = 0.02 [0.000, 0.045], *p* > 0.01; SRMR = 0.01; AIC = 14260.60; BIC = 14468.57] fitted the data the best. Table 4 shows that all the items loaded significantly on their respective factors, with factor loadings exceeding the 0.40 threshold. The first factor was labeled “grateful appraisals,” the second factor “gratitude toward others,” and the third factor “intentional attitude of gratitude.” The Oblimin factorial correlation showed that all factors were strongly correlated (with a range of *r* between 0.59 and 0.66; *p* < 0.01).

### Competing Confirmatory Factor Analytical Measurement Models

Next, a theoretically informed competing measurement modeling strategy was employed to further explore the factorial validity of the WGS. Measured items were used as indicators for latent factors, no items were removed, and error terms were not permitted to correlate. Four measurement models were estimated and compared:

• **Model 1**: A single first-order factorial model was specified where all 10 items loaded directly on to a single factor called “Work Gratitude.”
• **Model 2**: A three first-order correlated factor model was estimated for the factors labeled “grateful appraisals” (comprised of three items: SWGS1, SWGS2, and SWGS6), “gratitude toward others” (comprised of four items: SWGS5, SWGS7, SWGS8, and SWGS12), and “intentional attitude of gratitude” (comprised of three items: SWGS9, SWGS10, and SWGS11).
• **Model 3**: A second-order factorial model comprised of the three first-order factors specified in the previous model was specified to directly load onto an overall “Work Gratitude” factor.
• **Model 4**: A bifactor model with one general Work Gratitude factor (on which all 10 items directly loaded) and three specific first-order factors (as mentioned in Model 2) was estimated.

The results, summarized in Table 5, showed that Models 2, 3, and 4 fitted the data best. Models 2 and 3 produced the same fit statistics [χ^2^_(625)_ = 133.21; *df* = 32; χ^2^/*df* = 4.16; CFI = 0.98; TLI = 0.97; RMSEA = 0.07 [0.059, 0.084]; SRMR = 0.07]. This is expected if the three factors fully represent a second-order factorial model. Further, the bifactor model, Model 4, fitted the data significantly better than the second-order factor model, Model 2 (Δχ^2^ = −86.26; Δ*df* = −7; χ^2^/*df* = −2.28; ΔCFI = 0.02; ΔTLI = 0.03; ΔRMSEA = −0.03; ΔSRMR = −0.02; ΔAIC = −72.26; ΔBIC = −41.29). Both models further fitted the data significantly better than Model 1. Therefore, only Model 3 and 4 was retained for further inspection and analysis.

**TABLE 5:** Competing confirmatory factor analytical models.

Model	Type	χ^2^	*df*	χ^2^/*df*	CFI	TLI	RMSEA		SRMR	AIC	BIC	aBIC	Meets criteria
Model 1	Single first-order factor model	1113.4	35	31.81	0.75	0.68	0.22	[0.212–0.235]	0.09	15316.6	15449.3	15354.07	No
Model 2	Three first-order factor model	133.21	32	4.16	0.98	0.97	**0.07**	[0.059–0.084]	0.04	14342.4	14488.4	14383.6	Yes
Model 3	Second-order factor model	133.21	32	4.16	0.98	0.97	**0.07**	[0.059–0.084]	0.04	14342.4	14488.4	14383.6	Yes
Model 4	Bifactor model	**46.95**	25	1.88	1.00	1.00	**0.04**	[0.020–0.054]	0.02	14270.10	14447.1	14320.10	Yes

*χ^2^ = Chi-square; df = degrees of freedom; TLI = Tucker-Lewis Index; CFI = Comparative Fit Index; RMSEA = Root Mean Square Error of Approximation [90% CI]; SRMR = Standardized Root Mean Square Residual; AIC = Akaike Information Criterion; BIC = Bayes Information Criterion; aBIC = Adjusted Bayes Information Criterion.*

*Bold: Non-significant p > 0.001.*

### Item Level Descriptive Statistics, Standardized Factor Loadings, and Internal Consistencies

As shown in Table 6, all items were normally distributed (skewness and kurtosis <2; Kim, 2013). Each item was clearly associated with the overall factor being assessed (CITC *r* > 0.30; Zijlmans et al., 2019), and each of the three sub-factors and overall work gratitude was reliable at both the upper- (ρ > 0.80; ω > 0.80) and lower- bound level of internal consistency (α > 0.70). For the bifactor model, both the Omega for the specific factors and the Hierarchical Omega for the general factor were higher than 0.80.

**TABLE 6:** Item level descriptive statistics, factor loadings and internal consistencies of the second-order factorial and bifactor models.

Factor	Item	Model 3–second order factorial model														Model 4–bifactor model												
																G_factor_				S_factor_									

		$\bar{\text{X}}$	σ	Skw	Kurt	CITC	λ	S.E.	*R* ^2^	δ	AVE	*ρ*	α	ω	Meets criteria	λ	S.E.	*R* ^2^	λ		S.E.	*R* ^2^	δ	IECV	ARPB	ECV	OmegaS	OmegaH	Meets criteria
**Grateful appraisal**											0.73	0.89	0.88	0.89	Yes											0.32	0.89	–	Yes
SWGS 1		5.72	1.09	−1.20	1.54	0.82	0.88	0.01	0.77	0.23	–	–	–	–	Yes	0.70	0.03	0.49	0.59		0.04	0.35	0.16	0.58	0.043	–	–	–	Yes
SWGS 2		5.42	1.28	−0.83	0.17	0.80	0.90	0.01	0.81	0.19	–	–	–	–	Yes	0.76	0.02	0.57	0.45		0.04	0.21	0.22	0.74	0.020	–	–	–	Yes
SWGS 6		5.80	1.03	−1.35	2.46	0.72	0.77	0.02	0.59	0.41	–	–	–	–	Yes	0.67	0.03	0.44	0.41		0.04	0.14	0.41	0.76	0.044	–	–	–	Yes
**Gratitude toward others**											0.67	0.89	0.88	0.88	Yes											0.36	0.90	–	Yes
SWGS 5		5.67	1.20	−1.16	1.47	0.71	0.76	0.02	0.57	0.43					Yes	0.55	0.03	0.30	0.54		0.04	0.29	0.41	0.51	0.155	–	–	–	Yes
SWGS 7		5.80	1.00	−0.99	0.99	0.80	0.88	0.01	0.77	0.24	–	–	–	–	Yes	0.68	0.03	0.47	0.55		0.04	0.30	0.24	0.61	0.097	–	–	–	Yes
SWGS 8		5.73	1.03	−1.02	1.17	0.81	0.89	0.01	0.79	0.21	–	–	–	–	Yes	0.68	0.03	0.46	0.59		0.03	0.35	0.19	0.57	0.109	–	–	–	Yes
SWGS 12		5.75	1.03	−0.96	1.08	0.67	0.74	0.02	0.55	0.45	–	–	–	–	Yes	0.73	0.03	0.53	0.40		0.04	0.06	0.41	0.90	0.025	–	–	–	Yes
**Intentional attitude of gratitude**																										0.31	0.89	–	Yes
SWGS 9		5.57	1.19	−1.13	1.73	0.73	0.77	0.02	0.59	0.41	0.72	0.88	0.88	0.88	Yes	0.61	0.03	0.38	0.49		0.04	0.24	0.38	0.61	0.096	–		–	Yes
SWGS 10		5.78	1.06	−0.85	0.71	0.80	0.93	0.01	0.86	0.14	–	–	–	–	Yes	0.81	0.02	0.66	0.42		0.04	0.17	0.17	0.79	0.014	–		–	Yes
SWGS 11		5.85	0.96	−0.96	1.26	0.79	0.84	0.02	0.71	0.29	–	–	–	–	Yes	0.70	0.03	0.49	0.51		0.04	0.26	0.25	0.65	0.053	–		–	Yes
**Work gratitude**		5.70	0.83	−0.62	0.22	–	–	–	–	–	0.68	0.82	0.82	0.82	Yes	–	–	–	–		–	–	–	–	–	0.67	–	0.82	Yes
Grateful appraisal		5.65	1.02	−0.96	0.80	–	0.82	0.02	0.67	0.33	–	–	–	–	Yes	–	–	–	–		–	–	–	–	–	–	–	–	Yes
Gratitude toward others		5.74	0.92	−0.76	0.60	–	0.79	0.03	0.62	0.38	–	–	–	–	Yes	–	–	–	–		–	–	–	–	–	–	–	–	Yes
Intentional attitude of gratitude		5.73	0.96	−0.77	0.26	–	0.86	0.02	0.74	0.26	–	–	–	–	Yes	–	–	–	–		–	–	–	–	–	–	–	–	Yes

$\bar{X}$, *Mean; σ, Standard deviation; Skw, Skewness; Kurt, Kurtosis; CICT, Corrected item total correlation; λ, Standardized factor loadings; S.E., Standard Error; R^2^, Variance; δ, Item Uniqueness; AVE, Average Variance Extracted; ρ, Composite Reliability; α, Cronbach’s Alpha; ω, McDonald’s Omega.*

For the second-order factorial model, Model 3, all items in each subscale loaded statistically significantly onto their respective *a priori* factors with standardized factor loadings ranging 0.74 to 0.93 (λ > 0.40; *p* < 0.01; Kline, 2010). Further, the AVE for both factors (and the overall Work Gratitude factor) was higher than the suggested 0.50 cutoff point (Kline, 2010). Further, all three first-order factors, grateful appraisals (λ = 0.82, SE = 0.02, *p* < 0.01), gratitude toward others (λ = 0.79, SE = 0.03, *p* < 0.01) and intentional attitude of gratitude (λ = 0.86, SE = 0.03, *p* < 0.01) loaded statistically significantly onto the second-order Work Gratitude factor.

For the bifactor factorial Model 4, similar trends were observed. All items loaded statistically significantly onto both the general and specific factors with standardized factor loadings ranging from 0.40 to 0.81 (λ > 0.40; *p* < 0.01; Kline, 2010). Additionally, the IECV for all items exceeded the 0.50 threshold (Stucky et al., 2013). Further, the ARPB was below 15% on each item, therefore, no item-related bias was evident (Rodriguez et al., 2016). The ECV for the general factor was larger than the suggested 0.50 threshold, however, those for the specific factors ranged from 0.31 to 0.36. This implies that the general factor for the WGS is more representative of overall Work Gratitude, than the individual factors. This lower level of ECV is still acceptable, given that the threshold for the General Factor exceeds the limits.

Therefore, both models showed excellent levels of measurement quality and were therefore retained for further analysis.

### Measurement Invariance and Mean Comparisons

Next, the factorial equivalence of both Model 3 and Model 4 was tested with respect to males vs. females. The results, summarized in Table 7, showed that all models fitted the data based on the criteria described in Table 3. Further, no statistically significant differences in χ^2^ as well as RMSEA (Δ < 0.015), SRMR (Δ < 0.015), CFI (< 0.01), TLI (<0.01), and χ^2^/*df* (<1) between the configural, metric, scalar, and strict invariance models could be established (Cheung and Rensvold, 2002; Wong and Wong, 2020). Therefore, both models showed to be invariant between genders and meaningful mean comparisons can be made.

**TABLE 7:** Measurement invariance for gender.

Model		χ^2^	*df*	χ^2^*/df*	CFI	TLI	RMSEA		SRMR	Model comparison	Δχ2	Δχ^2^*/df*	ΔCFI	ΔTLI	ΔRMSEA	ΔSRMR	Meets criteria
**Second-order factorial model**																	
M1	Configural invariance	165.16	64	2.58	0.98	0.97	0.07	[0.058–0.085]	0.04	–	–	–	–	–	–	–	Yes
M2	Metric invariance: first order	182.66	71	2.57	0.97	0.97	0.07	[0.059–0.084]	0.06	M2 vs. M1	17.50	−0.01	0.00	0.00	0.00	0.02	Yes
M3	Metric invariance: second order	186.56	73	2.56	0.97	0.97	0.07	[0.059–0.084]	0.07	M3 vs. M2	3.90	−0.02	0.00	0.00	0.00	0.01	Yes
M4	Scalar invariance: first order	197.88	80	2.47	0.97	0.97	0.07	[0.057–0.081]	0.08	M4 vs. M3	11.32	−0.08	0.00	0.00	0.00	0.00	Yes
M5	Scalar invariance: second order	202.29	82	2.47	0.97	0.97	0.07	[0.057–0.081]	0.08	M5 vs. M4	4.41	−0.01	0.00	0.00	0.00	0.00	Yes
**Bifactor model**																	
M1	Configural invariance	67.11	50	1.34	1.00	0.99	0.03	(0.000–0.053)	0.02	–	–	–	–	–	–	–	Yes
M2	Metric invariance	103.31	66	1.57	0.99	0.99	0.04	(0.026–0.058)	0.07	M2 vs. M1	36.20	0.22	−0.01	−0.01	0.01	0.05	Yes
M3	Scalar invariance	116.18	72	1.61	0.99	0.99	0.04	(0.029–0.059)	0.07	M3 vs. M2	12.87	0.05	0.00	0.00	0.00	0.00	Yes

*χ^2^ = Chi-square; df = degrees of freedom; TLI = Tucker-Lewis Index; CFI = Comparative Fit Index; RMSEA = Root Mean Square Error of Approximation [90% CI]; SRMR = Standardized Root Mean Square Residual.*

For Model 3’s first-order factors, the results showed that no differences between males and females could be found for grateful appraisals (Δ x̄ = −0.02; SE = 0.12; *p* = 0.87), gratitude toward others (Δ x̄ = 0.08; SE = 0.13; *p* = 0.50) or intentional attitude of gratitude (Δ x̄ = 0.18; SE = 0.12; *p* = 0.12). No mean differences for overall Work Gratitude between males and females could be established (Δ x̄ = 0.09; SE = 0.12; *p* = 0.44).

Similar results were present for the Bifactor Model 4. No mean differences between males and females could be found on the three specific factors: grateful appraisals (Δ x̄ = −0.19; SE = 0.28; *p* = 0.48), gratitude toward others (Δ x̄ = 0.02; SE = 0.19; *p* = 0.93) or intentional attitude of gratitude (Δ x̄ = 0.17; SE = 0.22; *p* = 0.44). No mean differences between genders on the general Work Gratitude could be established (Δ x̄ = 0.11; SE = 0.17; *p* = 0.53).

### Convergent Validity With the Trait Gratitude Scales (GQ6 and Gratitude, Resentment, and Appreciation Scale)

To establish convergent validity, the two best fitting factorial models of the WGS were entered into a measurement model with two closely associated other measures of gratitude. The GQ6 was estimated as a unidimensional factor, with all items loading directly onto an overall dispositional gratitude factor. Further, the GRAT scale was estimated as a multidimensional factor comprised of three first order factors namely simple appreciation. appreciation for others and lack of a sense of deprivation. All items were specified to load onto their prior theoretical factorial models.

The results in Table 8 showed that for the second-order factorial Model 3 and the two trait gratitude scales, the data fitted the model adequately [χ^2^_(601)_ = 1195.659; *df* = 452; χ^2^/*df* = 2.65; CFI = 0.90; TLI = 0.90; RMSEA = 0.05 [0.049, 0.056]; SRMR = 0.07]. Both gratitude factors were strongly (*r* > 0.50) and statistically significantly (*p* < 0.05) related to the WGS.

**TABLE 8:** Convergent validity with the GQ6 and GRAT.

	Relationships	Standardized				Validity established
		*r*	S.E	*t*-value	*p*	
Second order model	WGS ←→ GQ6	0.50	0.06	8.97	0.00	Yes
	WGS ←→ GRAT	0.69	0.03	20.26	0.00	Yes
Bifactor model	Grateful appraisals ←→ GQ6	0.15	0.07	2.33	0.02	Yes
	Gratitude toward others ←→ GQ6	0.33	0.07	4.69	0.00	Yes
	Intentional attitude of gratitude ←→ GQ6	0.18	0.07	2.46	0.01	Yes
	Work gratitude ←→ GQ6	0.34	0.07	5.18	0.00	Yes
	Grateful appraisals ←→ GRAT	−0.11	0.06	−1.73	0.08	No
	Gratitude toward others ←→ GRAT	0.37	0.06	6.30	0.00	Yes
	Intentional attitude of gratitude ←→ GRAT	0.19	0.07	2.66	0.01	Yes
	Work gratitude ←→ GRAT	0.57	0.06	10.37	0.00	Yes

*←→ = Correlation; r = correlation coefficient; S.E = Standard Error; p = statistical significance.*

For the bifactor Model 4 including the two trait gratitude scales, the data also fitted the model adequately [χ^2^_(601)_ = 1118.47; *df* = 440; χ^2^/*df* = 2.54; CFI = 0.91; TLI = 0.90; RMSEA = 0.05 [0.047, 0.054]; SRMR = 0.07]. The results showed that the general Work Gratitude factor was significantly related to both the GRAT (*r* = 0.57; *p* < 0.05) and the GQ6 (*r* = 0.34; *p* < 0.05). All the specific factors, excluding gratitude toward others (*r* = −0.11; *p* = 0.08), related statistically significantly to GQ6 and GRAT. However, these relationships ranged from small to marginal. The results imply that the second order factorial model seems to be better associated with the trait gratitude scales than the bifactor model.

### Concurrent and Discriminant Validity

To establish concurrent and discriminant validity, separate structural models were estimated for the second-order Model 3 and the bifactor Model 4, and CSES and PCQ-12. For concurrent validity, CSES was specified as a unidimensional construct, where all items loaded directly onto a single factor. PCQ-12 was specified as second-order factorial model comprised of four specific factors (hope, efficacy, resilience, and optimism). In both models, demographic factors were used to establish discriminant validity. For concurrent validity, the WGS was regressed on both CSES and the second order PsyCap factor. For discriminant validity, the demographic factors were specified to correlate with the WGS.

The results in Table 9 showed that the second-order factorial Model 3, with demographic factors, CSES and PCQ-12, fitted the data adequately [χ^2^_(607)_ = 1722.41; *df* = 652; χ^2^/*df* = 2.64; CFI = 0.90; TLI = 0.90; RMSEA = 0.05 [0.049, 0.055]; SRMR = 0.06]. The results showed that none of the demographic factors related significantly to Work Gratitude (*p* > 0.05). Further, Work Gratitude was positively and significantly related to CSES (β = 0.38; SE = 0.05; *p* < 0.05; *R*^2^ = 0.15) and PCQ-12 (β = 0.61; SE = 0.04; *p* < 0.05; *R*^2^ = 0.37). Both concurrent and discriminant validity could therefore be established for the second order factorial Model.

**TABLE 9:** Concurrent and discriminant validity.

Relationships	Type of validity	Standardized						Validity established
		β	*r*	S.E	*t*-value	*p*	*R* ^2^	
**Second order model**								
Work gratitude ←→ Gender	Discriminant	–	0.02	0.04	0.45	0.65	–	Yes
Work gratitude ←→ Age	Discriminant	–	0.06	0.04	1.34	0.18	–	Yes
Work gratitude ←→ Occupation	Discriminant	–	−0.09	0.04	−2.24	0.06	–	Yes
Work gratitude ←→ Tenure	Discriminant	–	0.01	0.04	0.21	0.83	–	Yes
Work gratitude → CSES	Concurrent	0.38	–	0.04	9.13	0.00	0.15	Yes
Work gratitude → PCQ-12	Concurrent	0.61	–	0.04	17.19	0.00	0.37	Yes
**Bifactor model**								
Grateful appraisals ←→ Gender	Discriminant	–	−0.14	0.07	−2.04	0.06	–	Yes
Gratitude toward others ←→ Gender	Discriminant	–	−0.07	0.06	−1.07	0.28	–	Yes
Intentional attitude of gratitude ←→ Gender	Discriminant	–	−0.02	0.07	−0.31	0.75	–	Yes
Work gratitude ←→Gender	Discriminant	–	0.09	0.05	1.69	0.09		Yes
Grateful appraisals ←→ Age	Discriminant	–	0.04	0.06	0.70	0.49	–	Yes
Gratitude toward others ←→ Age	Discriminant	–	0.03	0.06	0.62	0.54	–	Yes
Intentional attitude of gratitude ←→ Age	Discriminant	–	0.24	0.07	3.56	0.00	–	No
Work gratitude ←→ Age	Discriminant	–	−0.02	0.05	−0.35	0.73	–	Yes
Grateful appraisals ←→ Occupation	Discriminant	–	−0.11	0.07	−1.62	0.11	–	Yes
Gratitude toward others ←→ Occupation	Discriminant	–	−0.16	0.06	−2.60	0.01	–	No
Intentional attitude of gratitude ←→ Occupation	Discriminant	–	−0.07	0.06	−1.13	0.26	–	Yes
Work gratitude ←→ Occupation	Discriminant	–	−0.01	0.05	−0.25	0.81	–	Yes
Grateful appraisals ←→ Tenure	Discriminant	–	0.01	0.08	0.10	0.92	–	Yes
Gratitude toward others ←→ Tenure	Discriminant	–	0.11	0.06	1.86	0.06	–	Yes
Intentional attitude of gratitude ←→ Tenure	Discriminant	–	0.18	0.08	2.41	0.02	–	No
Work gratitude ←→ Tenure	Discriminant	–	−0.01	0.06	−0.23	0.82	–	Yes
Grateful appraisals ←→ CSES	Concurrent	0.50	–	0.08	6.28	0.00	0.40	Yes
Gratitude toward others ←→ CSES	Concurrent	0.21	–	0.08	2.71	0.01	0.40	Yes
Intentional attitude of gratitude ←→ CSES	Concurrent	0.30	–	0.09	3.25	0.00	0.40	Yes
Work gratitude ←→ CSES	Concurrent	0.14	–	0.06	2.24	0.03	0.40	Yes
Grateful appraisals ←→ PCQ-12	Concurrent	0.63	–	0.07	8.68	0.00	0.66	Yes
Gratitude toward others ←→ PCQ-12	Concurrent	0.25	–	0.07	3.72	0.00	0.66	Yes
Intentional attitude of gratitude ←→ PCQ-12	Concurrent	0.30	–	0.08	3.61	0.00	0.66	Yes
Work gratitude ←→ PCQ-12	Concurrent	0.34	–	0.06	5.54	0.00	0.66	Yes

*→ = Regression; ←→ = Correlation; β = Standardized Beta; r = correlation coefficient; S.E = Standard Error; p = statistical significance; R^2^ = Variance.*

For the Bifactor Model 4, with demographic factors, CSES and PCQ-12, the results showed that the model fitted the data adequately [χ^2^_(607)_ = 1358.03; *df* = 614; χ^2^/*df* = 2.22; CFI = 0.91; TLI = 0.90; RMSEA = 0.05 [0.041, 0.058]; SRMR = 0.05]. The results showed that most of the demographic variables were not associated with neither the general nor the specific factors of the WGS, with three exceptions. Age (*r* = 0.24, *p* < 0.05) and tenure (*r* = 0.18, *p* < 0.05) were significantly related to intentional attitude of gratitude. Occupation was related to gratitude toward others (*r* = −0.16, *p* < 0.05). Therefore, discriminant validity could not be established. However, concurrent validity for the bifactor Model 4 was established as all specific and general factors related positively and significantly to CSES and PCQ-12 (*p* < 0.05).

Therefore, the results show that only the second-order factorial model was supported to be both concurrently and discriminately valid.

## Discussion

Despite its numerous recognized benefits, to-date there are limited applications of gratitude in the workplace. Furthermore, current gratitude measures pose a number of challenges that hamper effective measurement of gratitude at work. This study sought to fill this gap by developing and evaluating a WGS. Integrating the extant gratitude literature, we defined work gratitude as “the intentional choice to engage in positive appraisals and feelings of thankfulness and appreciation toward the characteristics, situations, and people currently present in one’s work context.” We used a systematic, six-phased approach to determine the factorial validity, internal consistency, measurement invariance, concurrent, convergent and discriminant validity of the WGS. Furthermore, we compared first-order, second-order, and bifactor competing models for Work Gratitude.

The results supported a 10-item measure for a second-order factorial model of work gratitude comprised of three dimensions: “grateful appraisals” (three items), “gratitude toward others” (four items), and “intentional attitude of gratitude” (three items). This second-order factorial model showed significantly better model fit, measurement quality, internal consistency, measurement invariance in relation to gender, concurrent validity in relation to two existing dispositional gratitude scales (GQ-6; McCullough et al., 2002, and GRAT; Thomas and Watkins, 2003; Watkins et al., 2003), convergent validity in relation to core self-evaluations (Judge et al., 2003) and PsyCap (Luthans et al., 2007; Avey et al., 2011), and discriminant validity in relation to demographic factors (age, gender, occupation and tenure) than other *a priori* factorial permutations.

This study, therefore, supports work gratitude as a second-order construct with three underlying first-order dimensions. From this perspective, work gratitude is operationalized and measured as a function of (a) *grateful appraisals of work* (i.e., positive, cognitive appraisals of work characteristics and situations), (b) *gratitude toward others at work* (i.e., social appreciation toward the contributions of others at one’s work), and (c) *an intentional attitude of gratitude* (i.e., purposefully enumerating, enjoying, and being mindful of positive aspects of one’s work). The results showed that this multidimensional conceptualization of work gratitude is related to, yet empirically distinct from other existing gratitude measures. In line with the findings of Cain et al. (2019), this means that employees who are generally inclined to show gratitude toward others may or may not necessarily show feelings of gratitude at work, and those experiences of gratitude at work may not necessarily extend to other life domains. Thus, work gratitude is related to, but conceptually distinct from gratitude in other life domains, or gratitude in general.

This multidimensional measurement strategy is closely aligned with the gratitude literature (Watkins et al., 2003; Morgan et al., 2017), as well as the positive organizational literature (Youssef and Luthans, 2013; Youssef-Morgan and Luthans, 2013; Luthans and Youssef-Morgan, 2017). This three-dimensional self-other-environment characterization of gratitude is also consistent with established psychological frameworks such as social cognitive theory, where agentic actions occur at the intersection of self-reflection, observation and learning from others, and influencing while being simultaneously influenced by one’s environment (Bandura, 2001, 2012; Bandura and Locke, 2003).

Specifically, gratitude is not just a unidimensional, deterministic dispositional trait (McCullough et al., 2002), or a transient, momentary affective state (Spence et al., 2014). It involves intentional, cognitive, affective, social mechanisms through which one reflects upon, evaluates, and appreciates various aspects of a specific context, such as meaningful and enjoyable work experiences, as well as the contributions of leaders, mentors, and colleagues at work (Luthans et al., 2015). The agentic and intentional components of gratitude are essential. Here, an employee purposefully chooses to be mindful of these various positive aspects of work and react to them positively and gratefully, rather than taking them for granted. Lyubomirsky (2007) posits that about 50% of positivity is trait-based, and circumstances determine only 10%, but 40% is open to growth and development through one’s intentional choices, thoughts and actions. This malleability and intentionality of gratitude is particularly relevant in the work context. For example, widely recognized gratitude development interventions (Davis et al., 2016) can be beneficial and effective if applied in the workplace to promote well-being, prosocial behaviors, and other desirable work outcomes. With the contribution of the current study, employee levels of work gratitude can be regularly assessed, monitored, and targeted for short and effective training interventions to promote grateful appraisals, gratitude toward others, and intentional attitudes of gratitude in employees.

### Strengths and Limitations

Among the notable strengths of this study are sample size and the availability and utilization of highly relevant constructs with valid and reliable measures (GQ-6, GRAT, CSES, and PCQ-12) to facilitate item generation and assess the concurrent, convergent, and discriminant validity of the WGS. Another strength is the rigorous and systematic testing of competing models and the highly consistent results supporting the two-factor model.

An important limitation of this study is that the sample was drawn from an educational environment, namely a medium-sized school district in the United States. This may limit the generalizability of the findings to other contexts. More effort should be taken by future researchers to investigate the factorial structure of the instrument in other work-related contexts. Furthermore, the majority of participants were young, female, teachers, with short tenure, which limits the external validity of the findings. However, the positions of the participants still varied widely, resembling a wide range of jobs in other industries and professions. Males, older, and longer tenured participants were adequately represented. Measurement invariance was supported for gender, and there was sufficient variation in gender, age, tenure and occupation to support discriminant validity.

Another limitation is that this study did not test for predictive or incremental validity of the WGS. Future studies can utilize the WGS to predict meaningful work attitudes, behaviors, performance, and other important work outcomes. They can also incorporate established predictors of such outcomes to assess the incremental validity of the WGS.

### Future Directions

Future research should examine the WGS in a wide range of work contexts such as manufacturing, services, and non-profit organizations of varying sizes to establish external validity. Furthermore, gratitude and other character strengths and virtues (Peterson and Seligman, 2004) are perceived and expressed differently across cultures. Thus, the WGS should be examined in other countries and cultures beyond the United States.

The availability of a valid and reliable measure of work gratitude is an important step in expanding the gratitude literature and research to the work context, linking it to strategic workplace initiatives such as human resource selection and development, and utilizing it to promote desirable work outcomes such as employee productivity, wellbeing, and prosocial behaviors. In terms of practical implications, there are many recognized and easy-to-implement gratitude interventions, which can be readily implemented in the workplace. Developing gratitude can yield highly desirable prosocial behaviors (Ma et al., 2017), which can promote employee wellbeing and a positive organizational culture.

Finally, the development of a context-specific measure of gratitude is an important step in operationalizing, measuring, and developing gratitude in other important life domains. Specifically, the WGS can be easily adapted to other contexts, by replacing the word “work” with other contexts of interest. This approach is similar to adaptations of the PCQ-12 to measure PsyCap in a variety of contexts (e.g., academic PsyCap, Martínez et al., 2019, 2021; relationship and health PsyCap, Luthans et al., 2013). Future research should rigorously examine and evaluate the psychometric characteristics of such adaptations of the WGS, but the availability of a context-specific measure of gratitude offers a valuable starting point.

## Data Availability Statement

The original contributions presented in the study are included in the article/supplementary material, further inquiries can be directed to the corresponding author.

## Ethics Statement

The studies involving human participants were reviewed and approved by the Western IRB. The patients/participants provided their written informed consent to participate in this study.

## Author Contributions

CY-M was primarily responsible for the conceptual framework of the manuscript, as well as leading and integrating the work of the research team. LZ was primarily responsible for the data analysis. BA was primarily responsible for the data collection. All authors contributed to the article and approved the submitted version.

## Conflict of Interest

The authors declare that the research was conducted in the absence of any commercial or financial relationships that could be construed as a potential conflict of interest.

## Publisher’s Note

All claims expressed in this article are solely those of the authors and do not necessarily represent those of their affiliated organizations, or those of the publisher, the editors and the reviewers. Any product that may be evaluated in this article, or claim that may be made by its manufacturer, is not guaranteed or endorsed by the publisher.

## Appendix A: The work gratitude scale

**Instructions:** For the following set of questions, think about your current working environment. Consider, the nature and function of your work, your colleagues/peers and the role work plays in your life. Then indicate to what extent you agree with the following 10 questions.

**Table:**

No	Item	Strongly disagree	Disagree	Slightly disagree	Neutral	Slightly agree	Agree	Strongly agree
***Grateful appraisals***								
1	Right now, I have so much at work to be thankful for.	1	2	3	4	5	6	7
2	At this present time, if I had to list everything that I felt grateful for at work, it would be a very long list.	1	2	3	4	5	6	7
3	At the present time, life has been good to me at work.	1	2	3	4	5	6	7
***Gratitude toward others***								
4	Currently, I couldn’t have gotten where I am today at work without the help of many people.	1	2	3	4	5	6	7
5	Although I think it’s important to feel good about my current work accomplishments, I think that it’s also important to remember how others have contributed to my accomplishments.	1	2	3	4	5	6	7
6	Although I’m basically in control of my work at the present time, I can’t help but think about all those who have supported me and helped me along the way.	1	2	3	4	5	6	7
7	Right now, I feel deeply appreciative for the things others have done for me at work.	1	2	3	4	5	6	7
***Intentional attitude of gratitude***								
8	Currently, I think that it’s important to “Stop and smell the roses” as it pertains to my work.	1	2	3	4	5	6	7
9	Currently, I believe that it’s important to pause often to “count my blessings” at work.	1	2	3	4	5	6	7
10	Right now, I think it’s important to enjoy the simple things that pertain to my work.	1	2	3	4	5	6	7

								
**Scoring**	Create an average or “mean” score of the following items to create a score for each of the components of the GWS							
	1.**Grateful appraisals** = (Item 1 + Item 2 + Item 3)/3							
	2.**Gratitude toward others** = (Item 4 + Item 5 + Item 6 + Item 7)/4							
	3.**Intentional attitude of gratitude** = (Item 8 + Item 9 + Item 10)/3							
	To create an overall score of work gratitude, create an average score of the means for each of the aforementioned components							
	1.**Overall Gratitude at Work** = (Grateful appraisal + Gratitude toward others + Intentional attitude of gratitude)/3							
